# Operation for Fissure of the Soft and Hard Palate, with the Results of Twenty Four Cases

**Published:** 1848-06

**Authors:** 


*Operation for Fissure of the Soft and Hard Palate, with the Results of twenty four Cases.* By J. Mason Warren, M. D., one of the Surgeons to the Massachusett's General Hospital. Boston: David Clapp, printer. 1848.

This is an interesting memoir, by a gentleman whose experience, in the operations of which he treats, is unusual, and whose success is extraordinary, if not unequalled; since out of twenty-six cases, twenty-thee of which were complicated fissure of the hard palate, twenty-five have proved completely successful. Roux, we believe, succeeded with only one out of three.

Dr. Warren's operation is original in several points:

1st. He relieves both the fissure in the soft and hard palate in a single operation.

2d. In cases of wide cleft, he cuts through the posterior pillar of the soft palate, and thus allows the edges to be brought more easily into contact. A similar process was suggested by Ferguson, in 1845, but the cases of Warren had precedence.

3d. The patients are allowed liquid food in the quantity required, by which the sufferings of the patient are not only greatly diminished, but the dryness of the fauces being prevented, the violence of the inflammatory process is greatly abated.

In one case, after cutting the posterior pillar, a troublesome hemorrhage occurred. In all of the cases a small opening remained after the wound had healed at the angle of the fissure, but this was subsequently closed by repeated applications of the nitrate of silver, &c.

In the conclusion of the pamphlet is an appendix on the subject of "early operations for hare-lip," of which he is a strenuous advocate. Dr. H. has repeatedly operated when the child was only twenty-four hours old! He prefers interupted sutures to pins, and recommends a straight needle.

We commend the pamphlet to our readers. F. H. H.

